# Supplementary material for: DotMotif: an open-source tool for connectome subgraph isomorphism search and graph queries
Source: Sci Rep. 2021 Jun 22;11:13045. doi: 10.1038/s41598-021-91025-5 (PMC8219732; doi:10.1038/s41598-021-91025-5)
Supplement: Supplementary file 1 — Supplementary Information 1 [file 41598_2021_91025_MOESM1_ESM.pdf]

DotMotif: An open-source tool for connectome subgraph isomorphism search and graph queries

Jordan K. Matelsky[1,*], **Elizabeth P. Reilly**[1], **Erik C. Johnson**[1], **Jennifer Stiso**[2,3], **Danielle S. Bassett**[2,4,5,6,7,8], **Brock A. Wester**[1], **and William Gray-Roncal**[1,9,*]

[1]The Johns Hopkins University Applied Physics Laboratory, Laurel MD 20723, USA
[2]Department of Bioengineering, School of Engineering and Applied Science, University of Pennsylvania, Philadelphia, PA 19104, USA
[3]Neuroscience Graduate Group, Perelman School of Medicine, University of Pennsylvania, Philadelphia, PA 19104, USA
[4]Department of Electrical and Systems Engineering, School of Engineering and Applied Science, University of Pennsylvania, Philadelphia, PA 19104, USA
[5]Department of Neurology, Perelman School of Medicine, University of Pennsylvania, Philadelphia, PA 19104 USA
[6]Department of Physics and Astronomy, College of Arts and Sciences, University of Pennsylvania, Philadelphia, PA 19104, USA
[7]Department of Psychiatry, Perelman School of Medicine, University of Pennsylvania, Philadelphia, PA 19104 USA
[8]Santa Fe Institute, Santa Fe, NM 87501 USA
[9]Johns Hopkins University, Department of Computer Science, Baltimore, MD 21218 USA
[*]jordan.matelsky@jhuapl.edu, william.gray.roncal@jhuapl.edu

Supplemental Materials

Code for the DotMotif Python library, the example snippets in this paper, and all data and code for the experiments run in *Results* are available online at bossdb.org/tools/dotmotif.

Code and documentation for the improved subgraph isomorphism library is available online at bossdb.org/tools/grandiso, and the graph-library abstraction tool discussed in *Optimized interfaces for common graph libraries* is available at bossdb.org/tools/grand.
